# Study of Deep Vein Thrombosis Screening by using Ultrasound Doppler in Patients with Pelvic and Acetabulum Fractures Requiring Operative Intervention

**DOI:** 10.5704/MOJ.2203.008

**Published:** 2022-03

**Authors:** D Hadizie, YB Deyoi, WI Faisham, S Yahaya, SA Ghani, MR Ahmad-Mohd-Zain

**Affiliations:** Department of Orthopaedics, Universiti Sains Malaysia, Kubang Kerian, Malaysia

**Keywords:** pelvic fracture, acetabulum fracture, deep vein thrombosis, pulmonary embolism, doppler ultrasound

## Abstract

**Introduction:**

Pelvic and acetabulum fractures are commonly caused by high impact injuries, increasing the risk of patients developing thromboembolic diseases such as deep vein thrombosis (DVT) and pulmonary embolism (PE). Therefore, this study was performed to determine the incidence of lower extremity DVT in patients with pelvic and acetabulum fractures and the importance of preoperative screening with Doppler ultrasound prior to surgical intervention.

**Materials and methods:**

This retrospective study involved 78 patients with pelvic and acetabulum fractures requiring surgical intervention from January 2015 until December 2019. Patients who underwent surgical interventions were screened pre-operatively with Doppler ultrasound to detect lower limb DVT and later compared with the incidence of lower limb DVT post-operatively. Descriptive statistical analysis was performed using IBM SPSS Statistics Version 24.

**Results:**

The participants of this study consisted of 30.8% females and 69.2% males. Pre-operative screening with Doppler ultrasound showed that three patients (3.8%) were diagnosed with lower limb DVT, whereas one of them (1.3%) was symptomatic and diagnosed with PE. Postoperatively, one patient developed DVT, and one patient developed PE. Both patients were negative for DVT preoperatively.

**Conclusion:**

The incidence of DVT in patients with pelvic and acetabulum fractures requiring operative intervention was significant despite the initiation of mechanical and pharmacological thromboprophylaxis upon admission. Despite the low incidence of DVT in our study, it has a high impact on patients’ morbidity and mortality; thus, preoperative screening is important for early detection and the subsequent reduction of the risk of developing PE. The compulsory use of Doppler ultrasound of bilateral lower limbs as a part of pre-operative screening is highly recommended because it is cost-effective, efficient and readily available in most tertiary hospitals nationwide.

## Introduction

Venous thromboembolic disease encompasses deep vein thrombosis (DVT) and pulmonary embolism (PE). Patients with pelvic and acetabular fractures are commonly associated with high impact injury and other related injuries in the head, chest, abdomen and extremities. Coagulopathy is evident in 25% of trauma patients upon admission and is associated with shock and a subsequent 5-fold risk of mortality^1^. Furthermore, all or one of the components of Virchow’s triad of hypercoagulability, venous stasis and endothelial dysfunction may be disrupted after major trauma, increasing the risk of thrombosis development^2^.

Patients with unstable pelvic and acetabulum fractures require operative fixation to achieve fracture stability, promoting early mobilisation and reduce post-operative hospital stay. Prolonged immobilisation is also an added risk factor to the development of venous thromboembolic diseases. Thus, mechanical and/or pharmacological thromboprophylaxis administration is recommended for patients upon admission as precautionary measures. Pharmacological thromboprophylaxis can be administered immediately, provided the patient is hemodynamically stable. However, appropriate delays in pharmacological thromboprophylaxis administration may be considered for patients with active bleeding, coagulopathy, hemodynamic instability, solid organ injury, traumatic brain injury or spinal trauma^3^.

Lower limb DVT can lead to PE, which can be detrimental and potentially fatal. The importance of screening and surveillance for DVT pre-operatively should be emphasised under these circumstances, especially in detecting asymptomatic patients to allow the administration of appropriate treatment and prevent further morbidity and mortality. Therefore, this study was performed to determine the incidence of lower extremity DVT in patients with pelvic and acetabulum fractures and the importance of preoperative screening with Doppler ultrasound prior to surgical intervention.

## Materials and Methods

This retrospective study involved patients treated in a single tertiary centre and a referral centre for advanced trauma services from January 2015 until December 2019. Referral cases from nearby hospitals which do not provide specialised orthopaedic services for pelvic and acetabulum injuries were also included in this study.

The inclusion criteria for this present study were patients aged 18 years and above with pelvic and/or acetabulum fractures requiring operative intervention and had undergone screening with Doppler ultrasound prior to operative intervention. On the other hand, patients aged below 18year-old with open pelvic and acetabulum fractures, patients with pelvic and acetabulum fractures which were managed non-operatively, patients who did not undergo screening with Doppler ultrasound before the operative intervention, patients with associated vascular injuries of upper or lower extremities requiring emergency operative intervention, patients with complex pelvic injuries requiring immediate operative intervention, patients with the previous history of DVT and patients on anticoagulants prior to admission were excluded from this study.

The participants were classified based on pelvic or acetabulum fractures after reviewing plain radiographs, and computed tomography (CT) scans by an advanced trauma surgeon and a radiologist. The acetabulum was not limited to the socket part of the “ball and socket” hip joint but also included the bony masses that limit and support the acetabulum^4^. Acetabulum fractures were graded according to the Judet and Letournel classification that categorised acetabulum fractures into simple (elementary) and complex (associated) fractures. The five elementary fractures were posterior wall, posterior column, anterior wall, anterior column and transverse fractures, whereas the five associated fractures were a combination of simple fractures and classified into posterior wall and posterior column fractures, transverse posterior wall fractures, T-shaped fractures, anterior with posterior hemitransverse fractures, and bicolumnar fractures.

Pelvic fractures were those fractures that led to the disruption of the pelvic ring. Pelvic ring injuries could be caused by fractures or joint disruptions due to ligamentous injury, contributing to pelvic ring instability^5^. According to Young and Burgess’ classification, pelvic injuries were classified based on anterior-posterior compression, lateral compression, or vertical shear. In cases of combined pelvic and acetabulum injuries, classification was determined by the most predominant injury.

Scoring of the patients risk of developing DVT was unnecessary since they were already considered a high-risk group because of their pelvic and acetabulum fractures and immobilisation. In our centre, mechanical and pharmacological thromboprophylaxis were administered to all patients at day 1 of admission based on the Malaysian Clinical Practice Guidelines (CPG) for Prevention and Treatment of Venous Thromboembolism (2013)^6^. Mechanical thromboprophylaxis using compression stockings or ankle pumps were administered prior to operative intervention. Meanwhile, pharmacological thromboprophylaxis with either low molecular weight heparin (subcutaneous Enoxaparin 40mg daily) or unfractionated heparin (subcutaneous Heparin 5000 units twice daily) was given, provided there were no contraindications and patients were hemodynamically stable. The type of temporary immobilisation for these fractures depended on the timing of operative intervention. If operative intervention could be performed within 1 to 2 days from the date of admission, patients were put on skin traction. However, if operative intervention was done later, patients were put on skeletal traction.

Patients were screened pre-operatively via Doppler ultrasound for lower extremity DVT a day or 2 before planned operative date by medical officers in the Radiology department and verified by Radiologists. Veins in patients’ lower extremities were assessed for patency, compressibility and echogenic foci suggestive of thrombus. The location of thrombi detected was classified into proximal or distal DVT. Proximal DVT was located at the popliteal vein or proximally, and distal DVT was located distal to the popliteal veins. Patients with proximal DVT were referred to the interventional radiologist for inferior vena cava filter insertion and started on intravenous heparin infusion to treat DVT prior to operative intervention. Surgery was performed one day after inferior vena cava filter insertion.

On the other hand, patients with distal DVT were treated with intravenous heparin infusion, and Doppler ultrasound was repeated 5-7 days later to assess for residual DVT before operative intervention. However, if the thrombi migrated proximally, patients were subjected to inferior vena cava filter insertion prior to operative intervention. Patients were prepared for surgery once the DVT had resolved. Symptomatic patients with PE, diagnosed via Computed Tomography Pulmonary Angiogram (CTPA) were comanaged in the intensive care unit (ICU) by the anaesthetic and medical team and treated according to standard management for PE with subcutaneous Fondaparinox 7.5mg daily and low molecular weight heparin (subcutaneous Enoxaparin 1mg/kg twice daily) based on the Clinical Practice Guidelines (CPG) for prevention and treatment of venous thromboembolism (VTE) (2013). Computer Tomography Pulmonary Angiogram (CTPA) was repeated after one week for post-treatment re-evaluation before proceeding with operative intervention.

Patients were not subjected to DVT screening postoperatively and prescribed with anticoagulants until discharged. The preferred post-operative anticoagulant in our centre was subcutaneous Enoxaparin 40mg once daily, combined with mechanical thromboprophylaxis using compression stockings and ankle pump. In cases where patients were symptomatic of DVT or PE, they were assessed clinically based on Wells’ criteria, and further radiological investigations were conducted. Doppler ultrasound was performed for patients suspected of DVT, whereas PE was confirmed via Computed Tomography Pulmonary Angiogram (CTPA) and respective treatment commenced upon diagnosis. Upon discharge, patients were prescribed with oral anticoagulants (oral Rivaroxaban 10mg once daily) and follow-ups by the medical team. The oral anticoagulant was continued for 3 to 6 months based on the recommendation by Malaysian CPG for Venous Thromboembolism. For patients with additional risk of VTE such as male and positive family history of VTE, the treatment will be given beyond three months to prevent the recurrence. Similarly, patients with inferior vena cava filters were discharged with oral anticoagulants until the filter is removed. The filter was removed when the patient becomes eligible for anticoagulant treatment as early as possible to prevent complications of prolong usage of the filter.

Patients’ information was obtained from their medical records and Picture Archiving and Communication System (PACS), including demographic data such as age, gender, comorbidities and associated injuries, the duration from the time of injury to operative intervention, timing and results of Doppler ultrasound pre-surgery. Statistical analysis was performed using IBM SPSS Statistics Version 24 [US]. This study has been approved by our Human Research Ethics Committee.

## Results

A total of 138 patients with pelvic and acetabulum fractures were admitted to our centre from January 2015 until December 2019. However, only 78 patients, 24 females (30.8%) and 54 males (69.2%), fulfilled the inclusion criteria and thus, were included in this study (Table I). A total number of 60 patients were excluded for these reasons: 22 patients did not have Doppler ultrasound screening performed prior to surgical intervention, 15 patients were managed non-operatively because of a stable fracture pattern or patients with multiple comorbidities rendering them unfit for surgery, while the remaining 23 patients were excluded as their previous medical records could not be traced. The patients’ age ranged from 18 to 80 years old with a mean age of 30. Sixteen (20.5%) patients were diagnosed with pelvic fractures, while the other 62 (79.5%) were diagnosed with acetabulum fractures. Furthermore, 32.1% of patients were referred from neighbouring tertiary hospitals, whereas 67.9% were admitted directly to our centre.

**Table I:** Patients with pelvic and acetabulum fractures based on gender

	Pelvic fracture (%)	Acetabulum fracture (%)	Total (%)
Females	3 (3.8%)	21 (26.9 %)	24 (30.7%)
Males	13 (16.7%)	41 (52.6%)	54 (69.3%)
Total	16 (20.5%)	62 (79.5%)	78 (100.0%)

Three (3.8%) patients were diagnosed with lower limb DVT based on pre-operative screening with Doppler ultrasound as illustrated in Table II, consisting of one male and two female patients below the age of 60 years old. All patients sustained acetabulum fractures in the ipsilateral limb accompanied by the presence of thrombi, of which two patients had a concurrent injury over the ipsilateral limb. The average waiting time for surgery was 15 days, and one patient underwent blood transfusion intra-operatively. These findings are summarised in Table III.

**Table II:** Incidence of pre-operative DVT and PE in patients with pelvic and acetabulum fractures

Classification	Presence of DVT (%)	Presence of PE (%)	No DVT/PE (%)
Acetabulum fractures	3 (3.8%)	0 (0.0%)	59 (75.7%)
Pelvic fractures	0 (0.0%)	1 (1.3%)	15 (19.2%)

**Table III:** Patients with DVT during preoperative screening

No	Age	Gender	Acetabulum/pelvic fracture	Time to surgery (days)	Associated limb injuries (Y/N)	Symptoms for DVT (Y/N)	Location of thrombus/thrombi	Blood transfusion	Wells score
1	38	F	Right acetabulum fracture	9	N	N	Right common femoral vein	2 pints 2 packed cells intra-operatively	1
2	32	F	Left acetabulum fracture	5	Left PCL and ligament injury	N	Left popliteal vein	N	1
3	19	M	Left acetabulum fracture	16	Laceration wound over right knee	N	Left common femoral and superficial femoral vein	N	1

Abbreviations: M: Male, F: Female, Y: Yes, N: No

A patient (1.3%) who was negative for DVT during preoperative screening with Doppler ultrasound developed PE pre-operatively (patient was scheduled for operation the day after screening but could not proceed due to unavailable OT). This patient was treated with anticoagulant according to the Malaysian Clinical Practice Guidelines and underwent operative intervention 17 days after the diagnosis of PE was done. All patients with proximal DVT were referred to Interventional Radiologist for inferior vena cava filter insertion, and intravenous heparin was administered, followed by operative intervention at a mean of two days after insertion of inferior vena cava filter. None of these patients developed symptomatic PE post-operatively.

One patient who was negative for DVT pre-operatively developed DVT post-operatively, while another patient developed PE as illustrated in Table IV. They were detected based on symptoms and scoring with Wells’ criteria for DVT and PE and subsequently underwent Doppler ultrasound for DVT and CT pulmonary angiogram for PE. Both patients diagnosed with PE were non-fatal and discharged soon after. The mean duration from the time of injury to operative intervention was 12 ± 7.96 days though the duration ranged from 3 days to 43 days. The timing of Doppler ultrasound performed after injury was 10 ± 7.8 days. Doppler ultrasound of bilateral lower limbs was performed at a mean of 2 ± 2.31 days prior to operative intervention.

**Table IV:** Proportion of patients negative for DVT preoperatively developing DVT and PE post-operatively

Classification	No DVT pre-operative (n = 74)	DVT post-operative (%)	PE Post-operative (%)
Pelvic fractures	15 (20.3%)	0 (0.0%)	0 (0.0%)
Acetabulum fractures	59 (79.7%)	1 (1.7%)	1 (1.7%)

## Discussion

The incidence of DVT in 2019 ranged from 3.0% -30.5% in pelvic and acetabulum fractures even with the commencement of anticoagulation^7^. In reality, this incidence is much higher and can reach up to 61.0%^8^. Results of the present study are comparable to that of Fishmann *et al*^8^, who reported a 6.0% incidence of pre-operative DVT in patients with pelvic and acetabular fractures. Meanwhile, Wang *et al*^7^ studied 110 patients with pelvic and acetabulum fractures who were started on mechanical and chemical thromboprophylaxis post-injury. DVT was detected in 29.1% of patients, of which 19.1% had proximal DVT, and 11.0% had distal DVT, while the other 2.7% developed PE. Conversely, the incidence was higher in the present study because of the inclusion of patients with VTE detected both pre-operative and post-operatively.

A retrospective study was conducted by Sen *et al*^9^ in patients with pelvic and acetabulum fractures in the Indian population who found 26.8% of patients had radiological evidence of VTE, of which six patients had clinical evidence of VTE while the rest were asymptomatic. Other Asian countries such as Korea and Japan reported VTE incidence of 33.7% and 41.3%, respectively^10,11^. These studies showed that despite the thromboprophylaxis commencement, patients were still susceptible to DVT; thus, preventive measures were needed to reduce the incidence of DVT and, subsequently, risk of PE development.

Borer *et al*^12^ conducted a retrospective study to compare the prevalence of PE in patients with closed pelvic and acetabulum with or without DVT screening. It was found that there was no difference in the rate of PE with or without screening for DVT as most patients had negative preoperative scans and a 0.3% rate of PE fatalities. Furthermore, Steele *et al*^13^ performed pre-operative screening only in patients with pelvic and acetabulum fractures transferred from referral hospitals seven days or more post-trauma and for all patients post-operatively with Duplex scanning but reported one fatal PE (confirmed by autopsy) before the scheduled Duplex scan. Thus, although pre-operative screening may not significantly reduce the incidence of PE, it is important to prevent the development of fatal PE. Meanwhile, Wang *et al*^7^ studied risk factors associated with DVT in pelvic and acetabular fractures, to which they concluded that patients older than 60 years, patients with associated injuries, time from injury to surgery of more than two weeks were associated with a higher risk of developing DVT.

Moreover, the rate of DVT is higher in acetabulum fractures compared to pelvic fractures. In the present study, all three patients sustained acetabulum fractures. Apart from the trauma and presence of hypercoagulability, manipulation during surgery can further increase the risk of developing DVT^14^, as reflected in patients developing DVT postoperatively despite negative pre-operative Doppler ultrasound findings. Trauma patients are highly susceptible to developing VTE due to their injury, rendering them in a hypercoagulable state. Wells’ criteria is a widely used tool to assess patients with the likelihood of developing DVT^15^. Nevertheless, Greenfield *et al* has upgraded the system by developing the Risk Assessment Profile to stratify trauma patients at risk of acquiring thromboembolic diseases based on age, underlying condition, iatrogenic and injury-related factors. Based on this Risk Assessment Profile, the pelvic fracture contributes to the risk of developing DVT^16^.

The timing for VTE screening varied between studies. Moed *et al*^17^ performed a prospective study on 229 patients on sequential Duplex ultrasound screening for DVT in asymptomatic patients with pelvic, and acetabulum fractures treated operatively. The screening was done pre-operatively, post-operatively and 24 hours before discharge. As a result, 35 patients (15.0%) were diagnosed with asymptomatic proximal DVT, where 7.0% were diagnosed pre-operatively, and 8.0% were diagnosed post-operatively. There was a possibility of missed proximal DVT pre-operatively, which was only detected post-operatively or thrombus developing while the patient was undergoing surgery. However, it was concluded that sequential screening did not reduce the risk of patients developing PE and may not be feasible, especially in patients with multiple traumas. Thus, Montgomery *et al*^18^ recommended pre-operative screening with compression Duplex ultrasound, especially in delayed pelvic fixation.

The gold standard for screening of lower limb DVT is contrast venography^19^, but it has limitations, including patient discomfort, high cost, and potential complications, thus making it a less popular choice of modality. It has prompted multiple studies to opt for other imaging modalities to screen for DVT. For instance, magnetic resonance venography is less invasive, does not require contrast in limbs with no implants inserted and is superior in detecting pelvic and proximal DVT^20^. In addition, Stannard *et al*^21^ reported that ultrasound had a 77.0% false-negative rate in detecting pelvic vein thrombosis compared to magnetic resonance venography, which had a 40.0% falsenegative rate. On top of that, Zhang *et al*^22^ conducted a metaanalysis to determine the diagnostic accuracy of ultrasound in detecting DVT in asymptomatic patients (no signs and symptoms of DVT) than that of venography. A subgroup analysis was done between pure compression technique, pure colour/Doppler technique and a combination of compression and colour/Doppler technique and their sensitivities were reported to be 43.0%, 58.0%, and 61.0%, respectively. Therefore, it was concluded that even though ultrasound was deemed useful in diagnosing DVT, it has a low sensitivity and high specificity as a screening tool for asymptomatic patients, who experienced a rate of missed diagnosis of lower-limb DVT of up to approximately 50.0%. In contrast, Shahzaad *et al*^19^ showed that colour Doppler ultrasonography had 99.0% sensitivity and 80.0% specificity in detecting DVT compared to venography. Based on this study, venography had a positive predictive value of 99.0% and a negative predictive value of 80.0% with a 99.0% sensitivity, 80.0% specificity and 98.0% accuracy. Despite its advantages, Doppler ultrasound has its limitations in detecting pelvic DVT. A recommended alternative would be ultrasonography to screen for DVT as it is safe, noninvasive, cost-effective and efficient. Other screening modalities may not be readily available, and time constraints in obtaining scan dates may further delay operative intervention. Therefore, Doppler ultrasound continues to be the most accessible modality for DVT screening^23^ since it is available in most local tertiary hospitals and less timeconsuming. Furthermore, it is a feasible option for preoperative screening in all patients with pelvic and acetabulum fractures.

The period from the point of injury to operative intervention varies widely due to several factors, although patients were operated within a mean of 12 days after injury. For example, most patients admitted had other associated life-threatening injuries that needed to be addressed immediately. Furthermore, some patients referred from other centres were treated for other associated injuries before being transferred to us, hence the delay in managing the pelvic and acetabulum injury. Moreover, financial constraints related to the payment of implants required for operative intervention may contribute to the delay in operative intervention.

In addition, the choice of chemical thromboprophylaxis varied between studies. Steele *et al*^13^ administered low molecular weight heparin in patients with pelvic and acetabulum fractures planned for operative intervention and compared the timing of administering chemical thromboprophylaxis, provided that haemodynamic stability was achieved. Upon comparison of the initiation of thromboprophylaxis within 24 hours of injury and after 24 hours of injury, it was found that the incidence of proximal DVT was significantly lower (3.0%) in patients administered with thromboprophylaxis within 24 hours compared to those provided after 24 hours (22.0%).

Several limitations were identified in this study. First, the small sample size may be attributed to the low incidence of DVT and PE in our studies. This is a retrospective study design, and many patients were excluded as they did not fulfil the inclusion criteria. Though there were multiple sources of information on patients’ data, there may be patients who were missed and not included in this present study. Furthermore, routine Doppler ultrasound screening of lower extremity DVT for patients with pelvic and acetabulum fractures planned for operative intervention only began over the last five years in our centre. Prior to this, most patients underwent operative intervention without screening unless they were deemed to be of high risk of developing VTE. Moreover, there were patients with stable pelvic and acetabular fractures, who were managed nonoperatively and thus, no screening was done for this group of patients unless they were symptomatic. The use of Doppler ultrasound as a screening modality is operator dependent, there is also a possibility that small thrombus may not be detected pre-operatively. Therefore, prospective studies with a larger sample size should be carried out to better ascertain DVT incidence in pelvic and acetabular fractures in the local population. Additionally, post-operative screening can be implemented to detect patients asymptomatic for DVT and assess response to treatment in patients diagnosed with DVT pre-operatively. Finally, Elnahal *et al*^24^ recommended preoperative and post-operative screening since they discovered that none of their patients with proximal DVT developed symptomatic PE.

In our centre, all patients with unstable pelvic and acetabulum fractures planned for operative intervention in more than 24 hours after injury will undergo Doppler ultrasound of bilateral lower limbs at least a day prior or on the day of operative intervention. This is an exception for cases which require urgent surgery such as open pelvic and acetabulum fractures requiring debridement or fractures involving vascular injury.

## Conclusion

In summary, the incidence of DVT in patients with pelvic and acetabulum fractures requiring operative intervention is significant in the present study, despite the initiation of mechanical and pharmacological thromboprophylaxis upon admission. Nevertheless, despite the low DVT incidence in this study, it has a high impact on patients’ morbidity and mortality; thus, pre-operative screening is important for early detection and subsequently reduces the risk of PE development. In addition, Doppler ultrasound of bilateral lower limbs is highly recommended as part of the compulsory pre-operative screening as it is cost-effective, efficient, and readily available in most tertiary hospitals in the country.
